# Health Promoting Sports Club in Practice: A Controlled Evaluation of the GAA Healthy Club Project

**DOI:** 10.3390/ijerph18094786

**Published:** 2021-04-30

**Authors:** Aoife Lane, Niamh Murphy, Colin Regan, David Callaghan

**Affiliations:** 1 SHE Research Group, Department of Sport and Health Sciences, Athlone Institute of Technology, N37HD68 Athlone, Ireland; 2 Centre for Health Behaviour Research, Department of Sport and Exercise Science, Waterford Institute of Technology, X91K0EK Waterford, Ireland; nmurphy@wit.ie; 3 Community and Health Department, Gaelic Athletic Association, D03P6K7 Dublin, Ireland; colin.regan@gaa.ie; 4 Sport Ireland, D15F2CC Dublin, Ireland; dcallaghan@sportireland.ie

**Keywords:** settings, sports club, health promotion, scaling up, systems thinking

## Abstract

Sport is a developing setting and a relevant system in health promotion but there are few examples of settings-based initiatives and systems thinking in sport. The Gaelic Athletic Association (GAA) Healthy Club Project (HCP) adopts a settings approach delivered through and by grassroots clubs who respond to local needs while working within a national support system. The aim of this evaluation was to assess and describe the health promotion impact and experience of the HCP. Healthy Clubs (*n* = 23) and Control Clubs (*n* = 10) completed a Healthy Club Questionnaire at the start and end of the 20-month HCP and Healthy Clubs took part in focus group discussions. Healthy Clubs, using the structures of the HCP, a commitment to health and community engagement, demonstrated a significant improvement in their overall orientation to health promotion, which was not apparent in Control Clubs. The health promotion message is pervading into many aspects of the GAA club apart from that which relates to the day to day business of coaching and providing physical activity for all. The HCP represents health promotion activity embedded within and across systems, with further development and evaluation recommended to measure delivery and impact at the individual level, organisational, and wider societal levels.

## 1. Introduction

Settings are places and social contexts where people live and work, and where environmental, organisational and personal factors interact to impact health [1,2,3,4]. Making these settings more supportive of population health is a long-held principle of health promotion [5]. Settings-based initiatives are characterised by organisational-level change, top-down leadership and bottom-up empowerment as well as a synergy between the agenda of the setting and public health [1]. However, most interventions in community-based settings have focused on providing health promotion at a singular level to individuals or groups, and have not considered broad changes to the setting itself [4]. Thus, the tendency is to adopt a passive rather than comprehensive approach to settings work [6]. This conflicts with our understanding of community settings as highly complex open systems within which polices are implemented, and where leverage points for enacting changes in the health of the community are often outside of the particular setting itself [7]. Systems thinking places ‘high value on understanding context and looking for connections between the parts, actors and processes of the system’ [8]. There is much in common with ecological models in health promotion, but systems thinking goes further by focusing on a system’s relationships and conditions and its processes of engagement and collaboration [7,9,10]. Recent Australian research has supported the intrinsic value of systems thinking for policymakers, when it is applied and policy-practice-orientated [11]. The focus should not be on documenting complex systems but on identifying ways to intervene in them. 

The sports club is evolving as a setting for health promotion, from the development of a theoretical background for the concept [12], to standards for a health promoting sports club [13] to a review of health promotion in youth sports [14], and finally to a framework for a health promoting sports club [15]. In this, social, environmental, economic, and cultural factors are described around three levels of the club setting: macro (overall policy and orientation of activities in a club), meso (officials and managers in the club), and micro (coaches and club members). Research activity has focused on describing the existing health promotion context in sports clubs [16,17,18,19,20,21], delivering healthy messages through the forum of sport [22,23,24,25,26], and identifying broader impacts of health promotion activity for sport in terms of enhancing participation and membership [18,27,28]. In sum, these studies indicated an orientation towards health among sports clubs’ playing members and coaches, the need for capacity building to make health promotion a sustainable activity for clubs, and support for sport-based health promotion activity. Intervention studies to assess impact on health behaviour are less common. A systematic review of health promotion strategies in sport offered no guidance for clubs due to a lack of well-designed studies [29]. Kokko and colleagues [30] presented case studies of sport club-based health promotion projects across Ireland, Australia, Belgium, Sweden, and Finland, with consensus on the value of this approach while acknowledging design limitations. A later review [31] identified only five randomised controlled trial designs out of 58 studies included in a review of the application of the settings approach in sports clubs. The majority of interventions were delivered at the intrapersonal level, targeting individual sports participants, with only two studies applying the settings approach across all levels of the social ecological model. These and others may be captured in a new systematic review [32] but, at this point, evidence suggests that the application of a broad settings approach in sport is minimal, and systems thinking is poorly developed in the field.

In Ireland, the Gaelic Athletic Association (GAA) Healthy Club Project (HCP) adopts a settings approach to health promotion in grassroots clubs [21,31] with examples of systems thinking. Firstly, in the absence of a full systems map for sport or health, the HCP reflects the national policy agenda for sport, physical activity, and health [33,34,35,36,37]. Secondly, the HCP evolved from ‘systems thinking’ across sport, health, education, political, and research sectors in Ireland, an example of activating the ‘co’ in coproduction [38]. Leverage for this approach comes from the GAA’s position as Ireland’s largest community and sporting organisation, promoting Gaelic games across Ireland and the world through a grassroots network of approximately 2000 volunteer-led clubs [39]. Club membership, attendance at events, and volunteering are at least three times higher for the GAA than any other team sports in Ireland [40], and 32% of Irish youth aged 12–18 years participate regularly in Gaelic games [41]. The GAA delivers many health benefits through the promotion of sport and physical activity, as well as broader volunteer-oriented social and economic health benefits through its club system, and it is led centrally by a national Community and Health section. In 2013, the GAA, in partnership with the national Health Service Executive (HSE), looked to harness the natural health orientation of the organisation and the health promotion potential of their club network to develop the HCP. A National Steering Group with representatives from the political, health, education, research, and sport sector developed a Healthy Club Framework (HCF) to reflect a systems-based approach within a club setting with upstream and downstream actions, including policy actions to influence environmental, social, and organisational change as well as individual level activity driven by clubs [42]. The HCF has four pillars, which are Plan, Club, Partners, and Activity:

- Plan relates to appointing a Healthy Club Officer, recruiting a Healthy Club Project Team, writing healthy club ideals into the club’s constitution and regulations and developing a policy, and action plans for health initiatives in the club.
- Club relates to the development of a physical and cultural environment that is health promoting.
- Partners involves partnerships with internal and external stakeholders and agencies to deliver health promotion.
- Activity includes interventions delivered on health issues such as physical activity, healthy eating, and social inclusion [42].

Phase 1 or the pilot phase of the HCP [21,43], implemented in 18 clubs, used a pre–post, intervention-group-only design. Data were collected using an adapted version of the Healthy Club Index [13] to assess the health promotion orientation of clubs. At baseline, clubs were positively oriented to, and already engaged in, some health promotion activity, while at follow-up, clubs showed improvement in their orientation towards, and action around, health. In total, 72 health promotion initiatives were run by 12 clubs across seven target areas, most commonly in the areas of emotional wellbeing, diet and nutrition, and alcohol/smoking. The majority of these initiatives included action on two to three of the four pillars of the HCF, and thus were categorised as low to medium impact [43]. Since Phase 1, health and wellbeing in the GAA has progressed in accordance with scaling up principles [44]. A National Healthy Club Co-Ordinator has been appointed, all clubs have been mandated to appoint Healthy Club Officers, regional Health and Wellbeing workgroups are in place, strategic partnerships with Healthy Ireland and the National Office for Suicide Prevention are established, and Irish Life (Assurance PLC) have committed to supporting the HCP through a corporate social responsibility investment. Finally, empirical support for the HCP [17,43,45,46] ensured a commitment to expand the healthy club model across the GAA in the 2018–2021 Strategic Plan [47]. This included scaling up to Phase 2 of the HCP to 60 clubs, Phase 3 and 4 for 150 clubs each, and a final Phase 5 to extend the HCP to all GAA clubs, with a commitment to maintain the fidelity of the initiative by empowering clubs to respond to local needs within a national support system. 

At this point, the GAA HCP represents, to the best of the authors’ knowledge, the only national sporting organisation-led sports club-based health promotion initiative. The evaluation of Phase 1 provided support for the impact of the HCP on the health promotion orientation of clubs, mitigated by an intervention-group-only design. For Phase 2, the GAA expressed a desire to enhance the evidence base for the HCP, which presented a novel opportunity to carry out a controlled evaluation of a feasible sports club-based health promotion initiative. The aim of the Phase 2 evaluation was to describe the impact and experience of the HCP on the health promotion activity and orientation of Healthy Clubs, in comparison to Control Clubs.

## 2. Materials and Methods

### 2.1. Sample 

Phase 2 of the HCP ran for 20 months from January 2016 to September 2017 and included Healthy Clubs (intervention) and Control Clubs. Clubs were invited to apply for Phase 2 using expression of interest forms that were distributed through a network of 32 communications officers, a national GAA club newsletter, and the GAA Community and Health website. One hundred clubs indicated their interest, and, subsequently, a GAA appointed subgroup of the National HCP Steering Committee screened all applicants and selected clubs to participate using a previously defined selection process. This considered (i) previous health promotion activity, (ii) capacity to participate in the programme, and (iii) inclusion of a variety of GAA club contexts in relation to size and location. In total, 44 clubs representing all 32 counties in Ireland were recruited to start Phase 2 of the HCP. Control Clubs (*n* = 27) were recruited through direct e-mails to club administrators and included clubs who applied for Phase 2 but were unsuccessful in their applications due to oversubscription of clubs with similar contexts. The latter facilitated purposeful recruitment of Control Clubs that fulfilled the selection criteria, therefore building an acceptable comparison group. Pending this process, further Control Clubs were recruited to complete matching on size and location. Ethical approval was granted by the WIT Research Ethics Committee.

### 2.2. Healthy Club Project

Healthy Clubs were invited to an Orientation Day. Important elements of participation in the HCP were presented to clubs.

- Clubs were advised that a Healthy Club Officer appointed to the Club Executive was an essential component of participation in this phase of the project, providing a governance structure to enable scaling up of the HCP [48]. Clubs were also recommended to recruit a Healthy Club Project Team with a minimum of four members.
- To enable policy development at local level, a GAA Healthy Club Statement was presented to clubs, as well as bespoke policies around physical activity, mental fitness, alcohol and substance abuse, and healthy eating.
- Clubs were encouraged to deliver initiatives on GAA-defined health topics (physical activity, healthy eating, mental fitness, community development, gambling, alcohol and drug education, and training and personal development) while also considering their own community needs.
- Clubs received training on the HCF and were supported to develop their own plans to deliver initiatives encompassing all four elements of the framework.

Clubs were given an overview of support and reporting structures in the HCP. Healthy Clubs were allocated to one of four regional working groups, which were organised primarily as a source of peer support to share experiences and ideas with other clubs and engage with the National Healthy Club Co-Ordinator. In addition, clubs were guided through an online portal where they reported health promotion activity and, finally, received an overview of the evaluation requirements. 

A mixed methods approach using quantitative and qualitative methods was used to assess the impact of the HCP on clubs and to understand the experiences of Healthy Club Officers involved in the leadership and delivery of the HCP. 

### 2.3. Quantitative Study: Questionnaires

#### 2.3.1. Design 

Healthy Club Officers in participating Healthy and Control Clubs completed a pre and post Healthy Club Questionnaire (HCQ) that described the physical environment, coaching context, community engagement, and membership of each club. At follow-up, Healthy Clubs also reported on the health promotion activity undertaken during the HCP. The main component of the HCQ was a Healthy Club Index (HCI), designed to assess the health promotion orientation of the club at the start and end of the HCP. The index was adapted from a validated tool (Health Promoting Sports Club Index- HPSC-I) [13] used to assess the health promotion orientation of Finnish sports clubs. A Delphi method developed 22 standards, divided across 4 sub-indices: Policy, Ideology, Practice, and Environment. Prior to Phase 1, the HCP Steering Committee and Evaluation team agreed to use this index, adapted to a GAA club setting. These revisions, which resulted in a 34-item index, included the following:

- Six of the eight items in the Policy index were retained. An original item on decision making was adapted to focus on the inclusion of health in the club constitution while a further original item on implementation was altered to a more specific focus on the fair distribution of playing pitches.
- The two Ideology items of ‘Everyone Plays’ and ‘Fair Play’ were adapted to refer to the GAA’s corresponding initiatives—Go Games and Respect.
- All of the original Practice items were retained.
- For the Environment index, there was one adaptation and two additions. The item on an environment free from intoxicants was adapted to ‘smoke-free’ and new items were added on respect for the referee and the provision of healthy food options following sporting activity in the club.
- A new sub-index was included to assess the Juvenile Coaching Environment in the GAA club. This was developed in response to challenges in the GAA around ensuring a participation-focused environment for youth players.

At baseline, 93% (*n* = 41) of Healthy Clubs and 96% (*n* = 26) of Control Clubs engaged with the evaluation process and completed the HCQ. Twenty-three Healthy Clubs and 10 Control Clubs completed the follow-up assessment, representing a response rate of 56% and 39%, respectively. This represents a considerable ‘drop off’ that is possibly related to the 20-month duration of the HCP. Control Clubs were likely very disengaged at the end of this period, and it is also possible that new Healthy Club Officers had been appointed in Control Clubs and were thus not familiar with their commitment to the HCP.

#### 2.3.2. Data Collection

The HCQ was distributed by the GAA Healthy Clubs Co-Ordinator to all Healthy Clubs, and by the Evaluation team to all Control Clubs via e-mail. Healthy Club Officers were encouraged to complete the questionnaire with assistance as needed from club officers or Healthy Club Project Team members. Baseline questionnaires were issued in April 2016 and submitted during the period April–May 2016. Follow-up questionnaires were distributed and completed in October–December 2017. 

#### 2.3.3. Data Analysis

Quantitative data were entered into Statistical Package for Social Sciences for analysis (SPSS 21.0). For the HCI, each of the 34 items in the index was scored on a 5-point Likert scale from 0 to 1. A score of 0 on any item signified this ‘does not represent the club at all’, while a score of 1 indicated this ‘represents the club very well’, which denoted overall that higher levels of health promotion activity were indicated by higher scores. The scores from each of the sub-indices (policy, ideology, practice, environment, and juvenile environment) were summed and an overall health promotion orientation score was generated for each club, ranging between 0 and 34. Clubs were classified as low, moderate, or high health promoting using categories used in Phase 1 of the HCP [21]. These were adapted from those developed by Kokko and colleagues to reflect the updated 34-item index used in the HCP [17]. Clubs who scored 23 or higher were deemed to be in the high health promoting category. Clubs scoring between 17 and 22.99 were categorised as moderately health promoting and clubs scoring less than 16.99 classified as low health promoting. A similar system was used to establish categories for each of the sub-indices. Within groups, changes in each sub-index and for the overall health promotion score were calculated using paired sample t-tests. Independent t-tests were used to compare between-group differences. Statistical significance was set at *p* < 0.05. Internal consistency of the overall index, measured using Cronbach’s Alpha, was high at both time points, 0.87 and 0.90, respectively. Scores for the sub-indices were acceptable at baseline, ranging between 0.76 and 0.79, while at follow-up, scores for policy, ideology, and practice were good, at 0.77–0.86, with environment and juvenile scores just meeting acceptability at 0.67. 

An impact rating scale of high impact, medium impact, and low impact was developed by the Evaluation Team to categorise the initiatives delivered by Healthy Clubs in the context of the four pillars of the HCF. Clubs described each initiative that they carried out, indicating if there was a policy or broader plan for the proposed action, detailing how the activity was impacting on the physical, social, coaching, playing, or cultural environment in the club, if it considered a partnership with local entities, and/or included an activity to address a health issue among individual or groups of participants. Clubs scored 1 for each pillar of the framework that they included, with a maximum score of 4 for each activity carried out. Clubs earned a rating of high impact if their initiative encompassed all four pillars of the framework. A medium impact rating was given for initiatives that comprised at least three pillars of the framework. Finally, a low impact rating was given when initiatives included up to two pillars of the HCF.

### 2.4. Qualitative Study: Focus Groups

#### 2.4.1. Design

Qualitative data were gathered using focus group discussions to support an understanding of the experience and impact of the HCP through the lens of Healthy Club Officers. Topic guides were developed by the research team, addressing concepts such as health promotion activity, success and challenges, and perceived impact of the HCP. Focus groups were moderated by a postgraduate student. Moderation included presenting topic areas through open questions, facilitating discussion, and inviting commentary from all participants. Clubs were encouraged to share their respective health promotion activity to illustrate their experience of the HCP. Focus groups were carried out in regional GAA units and Healthy Club Officers and/or a member of the Healthy Club Project Team from all participating Healthy Clubs was invited to attend their respective regional group session. 

#### 2.4.2. Data Collection

Four focus groups were carried out regionally with all Healthy Clubs at three stages throughout the HCP. The final round of focus groups, carried out in January 2017, was considered for this analysis. A total of 53 participants attended from 37 clubs, with 7 clubs absent across the four regional sessions. Two main topic areas (’successes and challenges’ and ‘perceived impact of the HCP’) were selected for this analysis to reflect the core aims of this paper, to describe the impact and experience of the HCP on health promotion activity and orientation of the club. 

#### 2.4.3. Data Analysis

The focus groups were transcribed verbatim by the researcher and compared against the original audio to ensure accuracy. Thematic analysis was carried out manually on the transcribed data and codes were identified to reflect the most salient and meaningful issues that arose around the selected topic areas. These were subsequently interpreted into three core themes based on patterns and recurring topics.

## 3. Results

### 3.1. Characteristics of Clubs

Characteristics of Healthy and Control Clubs who completed the baseline HCQ are presented in Table 1. There were no differences between clubs on membership, facilities and numbers of coaches, and similar practices around community engagement. Control Clubs were more likely to ensure that all coaches were accredited. There were no differences in the descriptive characteristics of Healthy and Control Clubs who responded at baseline only compared to those who engaged at both time points. 

### 3.2. Baseline and Follow-Up Analysis

Table 2 summarises the 34 individual items that contribute to each of the five sub-indices assessed in the HCQ across Healthy and Control Clubs who participated at both time points. 

All scores range between 0 and 1; 0 indicates that the factor does not describe the club at all and 1 indicates that it describes the club very well. At baseline, there were significant differences (*p* < 0.05) between Healthy and Control Clubs for the two ideology, one practice, and three each of the environment and juvenile environment items. 

Individual policy scores were among the lowest across all indicators at baseline for Healthy and Control Clubs but improved significantly (*p* < 0.05) at follow-up for Healthy Clubs only. Ideology items improved significantly (*p* < 0.05) in Healthy Clubs. Increases (*p* < 0.05) were apparent in Healthy Clubs for items in the practice and environment indices, in areas such as interaction with coaches and parents, providing health education opportunities and healthy food options, and developing smoke-free environments. Among Healthy Clubs, scores were higher (*p* < 0.05) at follow-up for items around selecting accredited and suitable coaches but scores for success not being defined by winning remained low and thus not reflective of the club. Moreover, among Healthy Clubs, there was an increase in the experience of barriers from parents and other clubs around implementing ‘everybody plays’. There were no significant (*p* > 0.05) changes in Control Clubs over time; scores remained low for policy items and high for ideology and environment items. Practice scores increased and there were no consistent trends for the juvenile environment items.

Table 3 collates the 34 individual items into their sub-indices and overall scores. At baseline, Control Clubs scored significantly higher (*p* < 0.05) for ideology and environment. Over time, there was a significant (*p* < 0.05) improvement in the overall health promotion orientation of Healthy Clubs and no change in Control Clubs (*p* > 0.05). There were equivalent improvements in each of the sub-indices for Healthy Clubs only. At follow-up, Healthy Clubs scored significantly (*p* < 0.05) higher than Control Clubs for policy and for overall health promotion, moving to high health promoting for both. Across other sub-indices, there was no difference at follow-up between Healthy and Control Clubs.

In addition, at follow-up, all Healthy Clubs and Control Clubs had appointed a Healthy Club Officer and all of the Healthy Clubs had developed Healthy Club Project Teams. At follow-up, all Healthy Clubs (*n* = 16) were represented on the Club Executive compared to 40% (*n* = 4) of Control Clubs. Finally, at follow-up, over 50% (*n* = 12) of Healthy Clubs and 20% (*n* = 2) Control Clubs noted that health and wellbeing issues were discussed at club meetings. 

### 3.3. Delivery of Initiatives

Healthy Clubs delivered initiatives across an average of four health areas, from a minimum of three and up to seven in one club (Table 4). Healthy Clubs were guided to include action on the four elements of the HCF: plan, partnership, activity, and club. Physical activity was the most common health focus for clubs and these initiatives were all rated medium or high impact, using three or all aspects of the HCF. There were examples of low-impact initiatives in all other areas, indicating that they included one or two pillars of the HCF, most commonly lacking a plan or partnership element. Medium rated initiatives generally lacked a plan or strategy. The most common partners were schools (16%) and various health/sport agencies (34%). The main target groups for initiatives were parents and families (28%), the wider community (25%), general club members (23%), and coaches/club officers (12%). 

### 3.4. Qualitative Data

Qualitative data confirmed support for the HCP around three core themes, (i) commitment to health, (ii) community engagement, and (iii) volunteer burden, with relevant codes italicised in the text. 

#### 3.4.1. Commitment to Health

The *natural affinity* of the GAA to health was uniformly expressed, reflected in observations that the ‘GAA is a lot more than games’ (Healthy Club Officer 1, Focus Group 1) and that health is a ‘natural partner to sport’ (Healthy Club Officer 1, Focus Group 2). It was consistently acknowledged that GAA clubs are in a unique position to affect people’s health behaviours and are subsequently being empowered to be active in the area of health and wellbeing in their community.

‘I think that health is everybody’s business, we’ve a responsibility to look after ourselves and to look after others’.

(Healthy Club Officer 2, Focus Group 1)

The overwhelming experience at the end of the HCP was that health was becoming a *priority for the club*.

‘I suppose we’re really happy in that after a couple of years of slogging away and, listening to other people that, kind of the health and well-being thing is really getting embedded into the club’.

(Healthy Club Officer 2, Focus Group 3)

At the same time, there were some challenges around ensuring that the commitment to health pervaded across the whole club and into the *core business* of the club. In all focus groups, there were various expressions that some groups were not integrated into the HCP. These included adult players, younger players, and men.

‘Participation from everybody would be a weakness, the buy in from everybody’.

(Healthy Club Officer 3, Focus Group 4)

The change in mindset around making health relevant in all club activity was most apparent for youth players.

‘In terms of the negative, it’s just like again, to reiterate what other people have said Sometimes it’s hard to get, if you win the final and stuff, the underage can be hard to get them to buy into it because they always want a party and stuff after and the parents would be getting pizza and stuff like that’.

(Healthy Club Officer 3, Focus Group 2)

#### 3.4.2. Community Engagement

It was noted in focus group discussions that clubs were opening up to the community and *attracting non-members* to the club.

‘Well there’s a mix, they’re not all members, no. The idea was to get all the community involved, you know....but the amount of people we didn’t know that were living in the area that actually came and got involved’.

(Healthy Club Officer 3, Focus Group 2)

‘We don’t even know half the people that were there (at healthy club event). So they’re not club members, they are community people’.

(Healthy Club Officer 2, Focus Group 4)

As a result, there was a sense that the HCP benefited how the club was perceived in the community, and that this stimulated a *renewed position* for the club in the community.

‘I think it’s improved the maybe, standing of the club in the community and made it more community orientated than just the club’.

(Healthy Club Officer 1, Focus Group 3)

‘It’s got a good name the Healthy Clubs, people want to be involved in it, want to be seen’.

(Healthy Club Officer 4, Focus Group 2)

#### 3.4.3. Volunteer Burden

The GAA, like many sporting organisations, is heavily dependent on volunteers to fulfil administration and coaching roles in clubs. Healthy Club Officers frequently noted the need to build and maintain a *network of people* working for the HCP.

‘No more than the rest of them, it’s trying to keep fresh volunteers coming in.’.

(Healthy Club Officer 4, Focus Group 2)

The HCP included an online portal where Healthy Clubs submitted and shared details of their ongoing activity. This proved a burden for Healthy Club Officers and there was a preference for a more *user friendly approach* to build a much valued community of practice among all Healthy Clubs.

‘The people that are doing these things are doing loads in the club, they don’t have loads of time to be logging on to these things everyday’.

(Healthy Club Officer 2, Focus Group 4)

‘The portal was meant to be supporting us and I think we’re missing a lot of what is going on and we could be supporting each other with a whatsapp group’.

(Healthy Club Officer 5, Focus Group 2)

## 4. Discussion

At the end of the HCP, Healthy Clubs had moved from moderate to high health promoting, and demonstrated significant improvements in all domains of the healthy club index. Healthy Clubs moved from low to high for policy, and importantly, all became connected with the Club Executive at follow-up. Control Clubs scored low for policy at both time points. Healthy Clubs also demonstrated improvements over time for ideology and environment scores, moving to parity with Control Clubs, who scored higher for these items at baseline. Practice scores increased but remained in the moderate category, and similar to Control Clubs. Although statistically significant, improvement for Healthy Clubs was least notable across the juvenile environment items, with clubs again just managing to move to parity with Control Clubs. While all clubs appear committed to embedding an ‘everybody plays’ policy (ideology), this was hindered by parents and other clubs, with scores for both of these indicators actually worsening over time. All clubs also scored poorly for their perceptions around the importance of success at youth level. Finally, Healthy Clubs were prolific in their delivery of initiatives, with 70–80% rated at least medium or high impact. Phase 2 of the HCP provided additional support for this programme, demonstrating impact through significant improvements in the overall health promotion orientation and particularly the policy activity of Healthy Clubs that were not apparent in Control Clubs. This presents a successful step in a rarely noted ‘scaling up’ story [49,50], moving beyond a pilot (Phase 1) to the development of a stable, funded, and efficacious programme, albeit with areas to further improve upon. In turn, this supports further development of the HCP to address these issues and reach more people over a sustained period of time [51]. 

At the outset of the HCP, participating clubs were classed, on average, as moderately health promoting. This confirms that while health is not always explicitly recognised in a sports setting, it is being practiced in clubs, providing leverage to start this work [15]. Using a similar assessment tool, a moderate orientation toward health promotion in youth sports clubs was noted in Finland [17]. In Belgium, youth sports clubs were mostly in the low health promoting category [19], while in France, 60% of clubs were classed as moderate–low health promoting [20]. These were all descriptive studies, with the HCP being the only project where health promotion orientation has been assessed over time following the delivery of an intervention. Previously, in Phase 1 of the HCP, intervention clubs moved from moderate to high health promoting at follow-up [43], while Phase 2 confirmed an improvement in the overall health promotion orientation of Healthy Clubs that was not reflected in Control Clubs. As per Phase 1 [17,19], the greatest impact of the HCP occurred in policy, as Healthy Clubs moved from low to high over the duration of the project. Similar to work in Australia [52] on state sporting bodies, the macro-level commitment in the GAA to establish clear governance and supporting structures around health promotion has had a strong, positive impact on the commitment to embedding health into club policy as well as scaffolding the implementation and scale up of the HCP in Phase 2 [44].

The four-year PICSAR programme in Australia [53] adopted a model like the HCP that favours a small number of sensitively applied, pre-determined actions mixed with a flexible, pragmatic, club- and community-defined approach. In Phase 2 of the HCP, national-level guidance and structures, particularly around governance, filtered down to grassroots clubs, who generated collective action around the framework presented. As a result, Healthy Clubs were active and showed significant improvements in the practice and environment domains of the HCQ as well as strong, positive perceptions of club culture and community engagement. Similar findings were noted in the Healthy Sporting Environments Demonstration Project (HSEDP), where the majority of clubs indicated that their club culture had changed for the better as a result of the project [54]. Notably, the HSEDP and other sports club-based interventions [55,56,57] have evaluated the impact of health promotion programmes on individual participants with mixed results, but this was beyond the scope of this analysis of the HCP. 

A breakthrough for settings work is apparent when health promotion activity aligns with the core business of the setting [15]. Similarly, creating supportive settings in sports clubs is related to the coaching and playing environment available for youth in sport so that they are safe, enjoyable, and focused on sustaining long-term participation [14]. In this approach, health is intertwined with organisational goals, and the value of the coach in health promotion activity with youth players is a priority. Despite this, current health promotion-related activity among coaches has been deemed narrow in scope and further coach education is required [26,28]. Meanwhile, it has been demonstrated that coach-created positive environments, where effort and personal improvement are championed, can positively impact enjoyment and motivation for sport, and subsequently improve physical activity [58]. Over the duration of the HCP, coaches were among the least targeted groups for activity. Healthy Clubs did become more oriented to recruit qualified and suitable coaches, and gave greater attention to coaches’ interaction skills. Healthy Clubs also reported improvements in the provision of healthy food options and considering health beyond sports performance. However, at baseline, all clubs indicated challenges in relation to prioritising maximum participation for all youth players and the HCP did not help Healthy Clubs to overcome this. This suggests a disconnect between coach education and the GAA ethos for participation in youth games and perhaps a failure to integrate health promotion thinking into the core on-field activity of playing sport. In turn, this presents a gap in the ‘micro’ layer of the HCP in the context of the health promoting sports club model [15]. The inactivity around maximising sports participation at youth level in GAA clubs, and the low–moderate rating of at least half of the Healthy Club initiatives, may be linked to the HCP model, of not prescribing initiatives for clubs. This approach reflects previous recommendations to prioritise activity and empowerment at the outset of an initiative, albeit with potential to increasingly guide implementation in light of long-term organisational aims [59,60], which may now be warranted. 

Engagement with the community in the HCP was strong, which is positive given that this is a feature of wider systems thinking. Indeed, at this point, the HCP has many characteristics of a working system and is a good example of a scaled up initiative. Key enablers for this include partnership with the health sector, a clear governance structure, local ownership, and community engagement [48]. At the same time, the GAA club is often a hub in a particular locality and could feasibly be a suitable setting for more integrated activity in communities and a greater public health impact [59]. It is time for the HCP to develop beyond the current scale to a more advanced systems model of practice, which will involve continued and broader, high-level organisational commitment as well as renewed consideration of the benefit to public health and as noted earlier, the core business of the institution. 

There are a number of limitations attached to this evaluation of the HCP. Firstly, clubs self-selected to take part in the study and therefore displayed an existing orientation towards health promotion. It is likely, given the community ethos, and previous health-related activity of the GAA, that all clubs undertake some health promotion practice, so, while generalisability within the GAA remains appropriate, generalisability to other sporting contexts should be considered carefully. It is accepted also that Control Clubs, through their engagement with the HCP application process, were disposed towards health promotion activity and, indeed, Control Clubs showed significantly higher scores for ideology and environment sub-indices at baseline and trended towards higher scores for practice and juvenile environment (Table 3). A selection bias to the intervention for clubs who had greater capacity to improve may have been apparent; however, overall health promotion scores and club characteristics were similar between groups at baseline. Subsequently, the HCP worked well to bring Healthy Clubs in line with Control Clubs for these four indices, which is a positive outcome, and a strong intervention effect was present for policy. In sum, the integration of Control Clubs into the design ultimately provides stronger empirical support for the HCP. Secondly, response rates were moderate to low, particularly for Control Clubs, which also impacts the generalisability of findings. There was no difference in the descriptive characteristics of Healthy and Control Clubs who responded at baseline and follow-up, which mitigates the notable drop off over the duration of the study. Thirdly, data were self-reported, and club experiences were those shared through the lens of the Healthy Club Officer only. Social desirability influences reporting and is likely to have been apparent in this national project by a country’s leading sporting and community organisation. Lastly, compiling and managing data was a challenge given the vast amount of activity undertaken and reported. In addition, the evaluation did not assess the impact of HCP activity at the individual level and did not adequately record the level of participation in the various activities undertaken, which limits the assessment of impact beyond the club level. However, the focus of Phase 2 of the HCP was to scale up from Phase 1 and assess the impact on club orientation towards health promotion, with an emphasis on empowerment by providing support and guidance without prescription and appreciating the context of clubs to work to their respective needs. A similar approach was used in a community initiative in Denmark [59] but with an added consideration of the delivery and evaluation of evidence informed initiatives, and individual-level monitoring [61]. Similarly, the social impact of community sports clubs in Australia has been carried out using club-based case studies and a survey of individual club members [62]. Future iterations of the HCP require a broader focus that includes explicitly (i) embedding health promotion ideals into the broader strategy and core activity of the organisation, (ii) targeting a public health impact, and, in time, (iii) developing a systems approach to addressing wider societal health issues. These require a long-term commitment to developing and evaluating the HCP over a 10–15-year period rather than a short-term focus around short-term health gains. This is reflected in a recommendation for settings to build resilience rather than efficiency and to embrace complexity and interconnectedness in a journey towards sustainable health promotion [59]. 

## 5. Conclusions

This novel evaluation of Phase 2 of the GAA HCP provides more robust support for the positive impact of the initiative on the health promotion orientation of clubs, particularly for policy and governance. The many elements of the HCP have nurtured a large amount of work to embed health promotion in clubs, which reflects the energy and affinity for this activity in GAA clubs. However, the focus and content of this work may require further consideration. Health promotion practice is least apparent in the day to day business of coaching and providing physical activity for all and does not yet pervade across all layers of the club. It is premature to determine Healthy Clubs as comprehensive health promoting sports clubs, but they are very much on the right end of the continuum in this point. Findings indicate that scaling up to Phase 3 should include the development of GAA-led initiatives specific to coaching and games activity while retaining a model with some independence for clubs to work to their respective needs. Further consideration of reporting and monitoring is necessary, particularly in relation to the impact of the HCP on the health and wellbeing of individual club and community members. Finally, while the GAA HCP is a very context-specific initiative, it provides a useful and relatively novel reference point for future sport-based health promotion activity. 

## Figures and Tables

**Table 1:** Descriptive characteristics of healthy and control clubs at baseline (*n* = 68).

	Healthy Clubs Phase II (*n* = 41)	Control (*n* = 27)
	Average	Average
Membership	468	502
Playing Pitches	2.2	2.2
Changing Rooms	4.2	3.4
Number of Youth Coaches	22	22
	**%**	**%**
Facility Rating (Excellent/Very Good)	75	82
Accredited Coaches	69	82
Support Community Events	67	74

**Table 2:** Sub-components of health promotion orientation for Healthy and Control Clubs.

	Healthy Clubs (*n* = 23) Average (0–1)		Control Clubs (*n* = 10) Average (0–1)	
	Baseline	Follow-Up	Baseline	Follow-Up
**Policy**				
The club’s regulations include a written section on wellbeing and/or health promotion/health education/healthy lifestyle	0.28	0.74 *	0.38	0.43 ^†^
The club’s regulations include a written policy on substance misuse (ASAP policy)	0.49	0.74 *	0.38	0.43 ^†^
Health and wellbeing ideals are written in the club’s constitution and regulations	0.21	0.67 *	0.25	0.25 ^†^
The club health promotion activities are evaluated in the Annual Report	0.27	0.77 *	0.20	0.28 ^†^
The club collaborates with other sports clubs and/or health professionals on health issues	0.40	0.75 *	0.63	0.50
The club assures that its subcommittees have agreed regulations and practices	0.49	0.73 *	0.53	0.53
Health promotion is part of the coaching practice	0.49	0.82	0.72	0.69
Training pitches and schedules are distributed fairly across all teams in the club	0.78	0.91 *	0.90	0.83
**Ideology**				
The club promotes the ‘Go Games’ principles	0.78	0.97 *	0.98 ^†^	0.95
The club promotes the ‘Respect Initiative’	0.71	0.93 *	0.90 ^†^	0.93
**Practice**				
The club’s Executive Committee discusses its regulations with coaches and parents at regular intervals	0.46	0.71 *	0.43	0.63
The club pays particular attention to coaches/instructors’ interaction skills	0.52	0.76 *	0.65	0.75
The club provides education on health issues or makes provisions for its members to receive such education	0.36	0.78 *	0.60	0.65
The club promotes individual growth and development	0.42	0.73 *	0.73 ^†^	0.78
Sports injuries are comprehensively dealt with (including the psychological effect of injury)	0.49	0.71 *	0.63	0.80
The club reviews and communicates treatment policies in the case of a sports injury	0.46	0.67 *	0.63	0.63
**Environment**				
The club assumes its fair share of responsibility for a safe sports environment (e.g., reviews the sports environment yearly)	0.73	0.85 *	0.85	0.78
The club provides a sports environment that is smoke-free during juvenile activities	0.52	0.84 *	0.78	0.85
Coaches and other officials give a good example through their own behaviour	0.67	0.88 *	0.88 ^†^	0.83
Respect for the referee is evident at all levels in the club (players, coaches, administrators)	0.57	0.82 *	0.83 ^†^	0.80
Possible conflicts (e.g., bullying) are monitored and dealt with	0.58	0.85 *	0.80 ^†^	0.85
In coaching, there is a health promoting element beyond sports performance	0.52	0.85 *	0.73	0.80
Healthy food options are made available following sports activities	0.34	0.71 *	0.55	0.53
**Juvenile Environment**				
All juvenile events are held in an alcohol-free environment	0.84	0.90	0.85	0.88
The club promotes maximum participation adopting an ‘every child gets a game’ policy	0.64	0.85 *	0.88 ^†^	0.83
The implementation of ‘everybody plays’ policy is not dependent on the importance of the competition	0.66	0.67	0.70	0.67
The implementation of ‘everybody plays’ policy is not hindered by parents’ expectations of success by winning	0.66	0.49 *	0.40 ^†^	0.62
The implementation of ‘everybody plays’ policy is not hindered by other clubs’ reluctance to adopt a similar approach	0.74	0.59 *	0.55 ^†^	0.60
The club measurement of success is not winning underage tournaments	0.40	0.40	0.50	0.35
The club does not perceive that success can only be achieved by having the best players on the pitch at all times	0.42	0.35	0.22	0.37
The club selects and approves coaches who have accredited coaching qualifications	0.64	0.78 *	0.75	0.68
The club specifically identifies suitable and qualified coaches for juvenile coaching positions	0.65	0.75	0.78	0.63
The club does not tolerate the use of bad language	0.57	0.75 *	0.70	0.68
The club enforces a fair play policy	0.62	0.83 *	0.83	0.83

* *p* < 0.05 Baseline vs. Follow-Up. ^†^ *p* < 0.05 Healthy Clubs vs. Control Clubs.

**Table 3:** Baseline and follow-up health promotion orientation of Healthy and Control Clubs.

	Healthy Clubs (*n* = 23)		Control Clubs (*n* = 10)	
	Baseline Score (Category)	Follow-Up Score (Category)	Baseline Score (Category)	Follow-Up Score (Category)
Policy Index (range 0–8.0)	3.41 (Low)	6.13 * (High)	3.95 (Low)	3.85 ^†^ (Low)
Ideology Index (range 0–2.0)	1.49 (High)	1.89 * (High)	1.88 ^†^ (High)	1.88 (High)
Practice Index (range 0–6.0)	2.71 (Moderate)	4.34 * (Moderate)	3.65 (Moderate)	4.23 (Moderate)
Environment Index (range 0–7.0)	3.92 (Moderate)	5.78 * (High)	5.40 ^†^ (High)	5.43 (High)
Juvenile Environment Index (range 0–11.0)	6.84 (Moderate)	7.36 * (Moderate)	7.16 (Moderate)	7.14 (Moderate)
Overall HP Index Score (range 0–34.0)	18.38 (Moderate)	25.52 * (High)	22.72 (Moderate)	22.51 ^†^ (Moderate)

* *p* < 0.05 Baseline vs. Follow-Up, ^†^ *p* < 0.05 Healthy Clubs vs. Control Clubs.

**Table 4:** Delivery and impact of HCP initiatives.

	Initiative Delivered (% Yes, *n*)	High Impact (%, *n*)	Medium Impact (%, *n*)	Low Impact (%, *n*)
Physical Activity	91 (20)	50 (10)	50 (10)	-
Healthy Eating	82 (18)	46 (8)	36 (7)	18 (3)
Mental Fitness	73 (16)	38 (6)	46 (7)	16 (3)
Training and Personal Development	68 (15)	48 (7)	33 (5)	19 (3)
Anti-Smoking	64 (14)	53 (7)	29 (4)	18 (3)
Community Development	59 (13)	33 (4)	50 (7)	17 (2)
Gambling, Alcohol, and Drug Education	36 (8) *	54 (4)	15 (1)	31 (3)
Anti-Bullying	18 (4) *	20 (1)	60 (2)	20 (2)

* numbers are small and should be treated with caution.

## Data Availability

Data are stored by the corresponding author.
